# Perceptions of Nigerian healthcare workers towards hand hygiene: a qualitative study

**DOI:** 10.11604/pamj.2020.36.204.19869

**Published:** 2020-07-22

**Authors:** Jude Nwaokenye, Sulaiman Lakoh, Julia Morgan

**Affiliations:** 1 University College Hospital, Ibadan, Nigeria,; 2 College of Medicine and Allied Health Sciences, University of Sierra Leone, Freetown, Sierra Leone,; 3 Connaught Hospital, University of Sierra Leone Teaching Hospitals Complex, Ministry of Health and Sanitation, Freetown, Sierra Leone,; 4 School of Public Health, University of Liverpool, Liverpool, United Kingdom

**Keywords:** Hand Hygiene, healthcare-associated Infections, alcohol-based hand rubs

## Abstract

**Introduction:**

hand hygiene (HH) is an effective measure to reduce healthcare-associated infections and the growing burden of antimicrobial resistance. There is a need to understand the perceptions of healthcare workers towards its practice and the use of alcohol-based hand rubs (ABHR) to make recommendations to promote HH. Our study aimed to explore the perceptions of Nigerian healthcare workers towards HH and the use and availability of ABHR to suggest potential interventions to improve its practice as qualitative evidence in this field is limited in Nigeria.

**Methods:**

a qualitative study design was utilized to understand the perceptions of healthcare workers towards HH and the use of ABHR at Adeoyo Maternity Hospital, Ibadan, Nigeria. Purposive sampling was used to recruit nineteen healthcare workers who were interviewed. Thematic content analysis was used to analyze the data generated.

**Results:**

five themes emerged including discrepancies in what constitutes HH practice as participants, motivation for HH practice, a good knowledge of timing as regards practice, barriers to good practice and evidence of poor practice.

**Conclusion:**

while many healthcare workers know about HH and self-reported compliance towards it seems to be high, knowledge gaps, lack of resources, absence of regulations and poor working conditions were impediments to the successful implementation of HH practices. We recommend that hospitals institute well-articulated HH regulations, continuous education and training of healthcare workers. Hospitals should also ensure adequate provision of resources for hand hygiene and institute a continuous monitoring and feedback program to evaluate compliance with regulations.

## Introduction

Despite supporting evidence on the importance of effective and consistent hand hygiene (HH) practices as simple measures with proven effectiveness in reducing the spread of healthcare associated infections (HAIs) [1,2], there is still poor compliance of healthcare workers (HCW) to hand hygiene practices in low- and middle-income countries (LMICs) [2]. Among the factors that promote poor HH practices in healthcare settings were lack of systems, poor infrastructure and limited behavioral change interventions [3,4]. A qualitative study in India identified interrupted supply of water, inadequate number of washbasins and the distance between patients and wash hand basins as barriers to HH practices [5]. Similar barriers as well as inadequate supply of alcohol-based hand rubs (ABHR) were even reported in a high resource setting [6]. In addition to the structural barriers, inadequate time [2], staff attitude, knowledge, education of HCW and workload issues [1] are functional impediments to effective HH practices. The ease of use of ABHR as a convenient alternative to antiseptic handwashing has been emphasized in several studies [2,3]. Due to its excellent invitro germicidal activity against most bacteria and fungi and less skin irritation, among other reasons, the World Health Organization (WHO) has recommended the use of ABHR in healthcare settings [4]. WHO recommends for HH practices to be part of routine healthcare using multimodal hand improvement strategy in 2009 in order to prevent the transmission of infections and reduce the growing problem of antimicrobial resistance [5]. Even with this recommendation, the capacity of institutions in LMICs to adopt and utilize guidelines on HH practices is low compared to high resource settings. A study in Nigeria found high rates of poor performance of HH among HCW [6]. As HCW play an active role in the transmission of infections [7], there is a need to understand their perceptions and motivations towards HH, as a precursor to enlightenment and behavioral change as well as creation of interventions to support their performance of HH. The public health significance of this study relates to the importance of the reduction of the global burden of highly resistant organisms within healthcare settings. Our study aimed to explore the perceptions of Nigerian HCW towards HH and the use and availability of ABHR in order to suggest potential interventions to improve HCW as qualitative evidence in this field is limited in Nigeria.

## Methods

**Study design:** a qualitative study design was employed to explore a phenomenon rather than begin an inquiry from a hypothetical set of facts, from first-hand accounts of the experiences of the participants in the field of interest in order to derive knowledge and understand meaning.

**Setting:** the setting of the study was Adeoyo Maternity Hospital (AMH) in Ibadan, Nigeria with a population of 5.5 million people. Founded in 1927, the hospital has a children outpatient department, obstetrics/gynaecology wards, a children´s ward, and a casualty department. Despite being a secondary hospital, approximately 3000 deliveries are carried out yearly [8].

**Sampling approach:** a purposeful convenience sampling method was adopted in the selection of participants. This study set out to have a sample size of 20, with each interview to last between 20 and 30 minutes. Only 19 interviews could be done because of an industrial action which greatly affected the number of participants that could be recruited.

**Participant recruitment:** a formal introduction was made to the head of the hospital and other senior doctors and nurses. Participants were HCW including nurses and doctors (18 years or older) in regular physical contact with patients who were not on vacation, or on an extended period of leave during the time of the interviews. Doctors and nurses in the obstetrics/gynaecology, paediatrics and the outpatient departments of the hospital were then approached and informed of the study by means of a participant information sheet (PIS). Appropriate verbal explanation of the content of the PIS was also provided to potential interviewees and dates were scheduled for the interviews. Participants were given time to understand the content of the PIS and to ask questions. Some of the interviews occurred on the same day that the participants were made aware of the study, others were scheduled according to their convenience.

**Data collection:** data collection was carried by means of semi-structured interviews to collect meaningful information on the experiences of interviewees about HH and the use AHBR. The interview explored four main areas: cues to performing hand hygiene, availability and use of ABHR, interpersonal dynamics and context. Before the interviews were conducted, informed consent was obtained from participants.19 key informant interviews were conducted on wards, clinics and offices between June and August 2017. The duration of the interviews ranged from 16 minutes to 38 minutes. The recording was done with a Samsung Galaxy tab which had an audio recording application and transcription was done verbatim by the research team. The total interview time for the research was 8 hours, 7 minutes and 39 seconds and a total of 58,593 words were transcribed verbatim from the audio-recorded interviews. Table 1 below is a summary of the specificities of the interviews.

**Table 1:** interview specificities

Participant(P)	Interview Duration	Location of Interview	Specificities
P1	32:52	Hospital lobby	Occasional interruptions
P2	16:35	Hospital lobby	Occasional interruptions
P3	22:31	Hospital lobby	Occasional interruptions
P4	26:18	Hospital lobby	Occasional interruptions
P5	28:22	Hospital lobby	Occasional interruptions
P6	23:42	Hospital ward	Occasional Interruptions
P7	35:07	Consulting room in a different hospital	Frequent interruptions
P8	33:05	Consulting room in a different hospital	Occasional interruptions
P9	30:20	Clinic	Occasional interruptions
P10	25:10	Office	Occasional interruptions
P11	23:42	Office	Occasional interruptions
P12	25:49	Office	Occasional interruptions
P13	28:39	Office	Occasional interruptions
P14	19:53	Office	Recording was broken into 2
P15	24:45	Common room	Minimal interruptions
P16	25:31	Office	A few interruptions
P17	15:22	Common room	No interruptions
P18	24:01	Office	A few interruptions
P19	25:55	Common room	No interruptions

Total interview time: 8 hours and 7 minutes and 39 seconds; total transcribed words: 58,593

**Ethical considerations:** ethical approvals were obtained from the Oyo State ethical review board and University of Liverpool ethical review boards. Participants were briefed on the nature of the research and what was expected of them and were given the opportunity to withdraw from the study anytime they wanted without any repercussions. They were assured of anonymity and confidentiality. Data was kept in a password protected computer hard drive and anonymity was ensured by replacing their names with a number in the transcripts.

**Data analysis:** data analysis was done using thematic analysis with data analysis roughly followed 6 phases; from familiarization of the data through transcription, generation of initial codes from the transcripts to generation of themes and producing results.

## Results

Key findings are summarized in Table 2. Socio-demographic characteristics of participants and the themes of analysis are summarized in Table 3and Table 4, respectively.

**Table 2:** key findings

Discrepancies in what constitutes hand hygiene
All participants rated themselves high in HH practice even though further talks suggested otherwise
There was a general knowledge of available options for HH, but many could not say which was more effective
All agreed on the place of ABHR in HH but there were differences of opinion in how it was to be used and most had no idea of the acceptable quantity to be used
**Motivation for hand hygiene practice**
Increase in awareness of hand hygiene and the influence disease outbreaks on practice
Prevention of infection and hand hygiene as a part of routine.
**A good knowledge of timing**
All participants had a good knowledge of timing of hand hygiene practice
**Barriers to practice**
Financial constraints, poor staff remuneration, absence of regulations, lack of amenities and workload pressures were all factors militating against hand hygiene practice
**Evidence of poor practice**

Certain knowledge gaps and practices were evidence of poor hand hygiene practice. These included occasional failures to perform hand hygiene, poor hand drying practices, and a lack of knowledge of, “My five moments of hand hygiene”

**Table 3:** study participants´ demographics

Participant	Age range	Gender	Profession	Recruitment
P1	35-40	M	Doctor	Professional contact
P2	30-35	F	Doctor	Professional contact
P3	30-35	F	Doctor	Professional contact
P4	35-40	F	Doctor	Professional contact
P5	30-35	F	Doctor	Professional contact
P6	35-40	M	Doctor	Professional contact
P7	35-40	M	Doctor	Professional contact
P8	35-40	M	Doctor	Professional contact
P9	40-45	M	Doctor	Professional contact
P10	40-45	F	Nurse	Professional contact
P11	45-50	F	Nurse	Professional contact
P12	45-50	F	Nurse	Professional contact
P13	40-45	F	Nurse	Professional contact
P14	25-30	F	Nurse	Professional contact
P15	45-50	M	Nurse	Professional contact
P16	35-40	F	Nurse	Professional contact
P17	25-30	F	Nurse	Professional contact
P18	35-40	F	Nurse	Professional contact
P19	50-55	F	Nurse	Professional contact

**Table 4:** themes and subthemes

Themes	Subthemes
**Discrepancies in what constitutes hand hygiene**	
	Good performance
	Options for hand hygiene
	When to use alcohol hand sanitizers
**Motivation for hand hygiene practice**	
	Increased awareness
	Influence of disease outbreaks
	Prevention of infection
	A part of routine
**Good knowledge of timing**	
	Hand hygiene before and after procedures
	Hand hygiene after de-gloving
	Hand hygiene after bodily fluid exposure
**Barriers to good practice**	
	Financial constraints
	Absence of regulations
	Lack of amenities
	Workload pressure
	Absence of alcohol hand sanitizers
**Evidence of poor practice**	
	Occasional failures to perform hand hygiene
	Poor practice in drying hands
	Lack of knowledge of “My Five moments of Hand hygiene”
	Poor knowledge of quantity of hand sanitizer needed for hand hygiene

### Theme 1: discrepancies in what constitutes hand hygiene

This theme comprises of several sub-themes including good HH performance, options for HH and use of ABHR.

**Good performance:** all the participants considered their practice of HH and that of the hospital staff in general, to be above average. One of the participants, while acknowledging that he could be carefree, still considered his adherence to be well above average. This high appraisal of the self-performance was, for many, higher than the perceived general rate of performance in the hospital, which was also often above average. Although all participants had been assured of confidentiality the body language of a few of them, especially the nurses, suggested a desire to give an impression that all was well. P11: *“...my personal one (rate of hand hygiene), I´ll say it´s hundred percent.”*

**Options for hand hygiene:** all participants had a general idea of the available options for HH, but there were differences in what these were. They all considered the use of soap and water and ABHR as options but did not have much to say about the effectiveness of one over the other. Also, very few talked about a distinction in the type of soaps to be used; antiseptic or plain. P1: *“The best way is to use soapy solution under running water...but in a situation where the water or soap are not available we can opt for hand sanitizers...Yes antiseptic soap is proper but here we don´t use it, I must confess we don´t use antiseptic soap here, it´s just ordinary liquid soap.”*

**The use of alcohol hand sanitizers:** there were different ideas about the role of ABHR in HH. There was an understanding on the part of some that it could be used in place of water if the hands were not very dirty, while others thought it was to be an adjunct to regular washing with soap and water and not necessarily to be used alone. P12: *“After washing we sanitize our hands. ”*Also worthy of note was the fact that most of the participants appeared not to have considered the importance of the quantity of ABHR which needed to be used in hand hygiene. Only P15, who was a nurse in the theatre, said the quantity should be enough to be rubbed up as far as the elbows.

### Theme 2: motivation for hand hygiene practice

Some inherent factors that seemed to encourage the practice were revealed in this theme and resulted in the following sub-themes.

**Increased awareness:** the recent increase in awareness of HH practice in the last couple of years was a subtheme that re-echoed from many of the participants as a cause of an increase in HH practice. A major factor responsible for this was the episodes of disease outbreaks in the country. P8: *“But I think awareness about hand hygiene has increased after that Ebola outbreak and in this hospital, I think, you want me to give you figure as well?”*

**Influence of disease outbreaks:** this subtheme was developed as responses from several participants showed lingering consciousness of the outbreaks of viral hemorrhagic fevers. The most notable example cited was the 2014 outbreak of ebola virus disease. This influence of disease outbreaks was seen in the practice of HH in general and in the use of ABHR in particular. Indeed, P6 had never known about ABHR before this outbreak and thought that it only came into use in its aftermath. P6: *“Well, the alcohol hand sanitizers...I know it came into (widespread use in) this country basically after the ebola outbreak...that´s when the federal and state governments made it available...for everybody, for all the hospitals in Oyo State.”*P9:”*...hand hygiene practice was, very low but, immediately after the epidemic of ebola, the level was high, presently it´s getting low again.”*

**Prevention of infection:** while all the participants were mindful of the role, they played in preventing the spread of infections to patients they attended to, some were also concerned about contracting infections from patients. Three of them expressed concerns of not transferring infections to their children at home. P10: *”I don´t want to transfer germs to my patient because they´re the main reason why I´m here in the first place. I want to protect myself, I want to protect my family.”*

**A part of routine:** HH was seen as an innate part of medical or nursing practice, having been learned during the training of the health care professionals. The participants felt it was something necessary in the routine of patient care. P11: *“In the profession you know before you do anything you have to wash your hands. It is something that is registered.”*

### Theme 3: good knowledge of timing

Developed as a direct response from questions that were asked, it was adjudged a theme because it tested participants knowledge of the WHO initiative, “My five moments of hand hygiene.”

**Hand hygiene before and after procedures:** all the participants agreed on the importance of HH practice after medical or nursing procedures as means of curtailing the spread of infections. P6: *“It´s important that at the start of seeing a patient hand hygiene is very essential. I think that just like I keep saying, in paediatrics before you touch a particular baby you go and wash your hands, after touching that baby you wash your hands whether that´s the only baby you´re seeing or that´s the last baby, wash your hands before, wash your hands after.”*

**Hand hygiene after de-gloving:** many of the participants considered HH after glove use as important. This subtheme was developed after repeatedly being seen among the participants. P1: *”It´s a policy here in this hospital before we examine any patient, we put on our gloves so after we have finished seeing the patient you remove the glove then you go ahead and wash both hands.”*

**Hand hygiene after bodily fluid exposure:** there was a unanimous agreement, on the side of participants, that HH after body fluid exposure was of utmost importance.

### Theme 4: barriers to practice

This theme was developed as participants expressed frustrations, they experienced in the workplace which made the practice of HH difficult. Four subthemes were developed under this theme.

**Financial constraints:** many complained of inadequate financing. This factor, which was seen not only in the area of hospital funding and procurement of resources but also in staff remuneration, was a common reason for poor practice. P6: *“A lot of things are wrong, people are not happy, no salaries...so these are the issues.”* P9: ”*We complain but the management say there´s no fund. They´re telling the truth. The amount of imprest the hospital is given has been halved. Even that half is not regular.”*

**Absence of regulations:** all the participants responded in the negative when asked about of the presence of HH regulations within the hospital. P15: *”In terms of policy? No.”*While many considered such regulations as realistic and did not mind its introduction, P4 did not think it would be easy for such to work without addressing other fundamental issues such as lack of amenities. P4: *”Well, it´s not really realistic in the sense that there are limitations to the rules, in each ward there is this handwashing procedure that is pasted on each wall. One of the things that affects that is that at times we might not have running water.”*

**Lack of amenities:** the sense of lack and disaffection with the work environment was portrayed in several ways such as frequent shortages in running water, power outages, a limited supply of ABHR and lack of disposable towels. P6: *”Well the rate in the hospital in general I would say well, it´s like 80%. Most of the time when you want to wash your hands and there´s no power supply to pump water, you get discouraged. So most of the time you just go away and probably assume that you are going to clean your hands when you get home and more so the alcohol hand sanitizers are not readily available in this environment, sometimes it´s even expensive and in a case whereby someone has not been paid, is that time you´ll go and use the little that you have to buy hand sanitizers?”*

**Workload pressure:** most considered the workload too strenuous to allow for adequate HH performance. This workload was expressed in both the number of patients´ participants had to attend to, the hospital being situated in a densely populated area and the limited number of staff there was to work. Participants often got so busy that they either forget or were unable to perform hand hygiene. P6: *”Well, most of the time, I see like 200 patients per day. I don´t know if it´s possible to wash your hands 200 times, so most of the time it´s not feasible. That´s the sincere truth. It´s either you´re finishing one examination, before you know it, patients they´re already queuing up and if you have to see 200 patients and one doctor that´s killing, so washing your hands 200 times is stressful.”*

### Theme 5: evidence of poor practice

Several instances of poor practice in relation to HH are discussed under two sub-themes below.

**Occasional failures to perform hand hygiene:** on several occasions there was a failure to perform HH. Sometimes participants felt it could be delayed as they felt the successive use of gloves could obviate the need for performance. P3: *”Well, I´m in the outpatient section, I see a lot of patients and for every patient I wear a pair of gloves so if I have to wear a pair of gloves stand up wash my hands, come back, see another patient stand up, it´s not easy, so most times I just keep changing the pair of gloves. For every patient I wear a pair of gloves.”* There was also the feeling that if a procedure was not strictly an aseptic one, there was really no need to perform HH. For example, the recording of vital signs such as pulse and temperature could be considered innocuous activities that do not require HH. P5: *”I think, if what you´re doing is not an aseptic procedure then you don´t have to wash your hands except if your hands are really dirty, .let´s say you´re coming from a place where not too long ago you washed your hands, you´ve not done anything with your hands, the next patient that comes in you want to examine the patient and you need to wear gloves to do that examination doesn´t mean that you have to wash your hands.”*

**Poor practice in drying hands:** all agreed that drying of the hands was an important part of HH when they were specifically asked and many felt that their practice of hand drying was inadequate. There was no adequate provision for hand drying even in places where a wash station was provided. The routine practice was to have a hand towel that was reused. Most were however loathed to admit to this practice of sharing towels and said they made use of their own personal hand towels why a few preferred to air dry their hands after washing. P8: *”In my practice here there are times...okay, I go around with my handkerchief as you can see I have...I have a number of them at home...I use them to dry my hands...in some places in the hospital some nurses or doctors provide handkerchiefs beside the wash hand basin. I don´t like it because people just come and use it, it may even cause more infection...so I will say dry your hands with a clean hand towel, preferably a personal one.”*

## Discussion

This study sought to understand the perceptions of Nigerian HCW towards HH and the use of ABHR. To the best of our knowledge, the study fills a gap in current research on the subject being the first qualitative study of its kind in the country. All the participants scored themselves high in HH performance. This high appraisal is in keeping with findings from previous studies which showed that healthcare workers often have a high estimation of their performance of HH and other infection control practices [9,10]. By reflecting on the information component information-motivation-behaviour (IMB) model, the findings show that none of the HCW felt that they had any knowledge deficits concerning HH. As the conversation unfolded however, knowledge gaps were often shown which replicated findings from Bello and colleagues [11]. An example of such was the little resolve shown by most participants concerning the obligate use of antiseptic soaps for HH. This was not surprising as the kind of soap usually supplied to the wards and clinics were ordinary liquid soaps. Handwashing with plain soap, while able to clean visibly soiled hands, has been shown to be unable to get rid of microbial contamination [12]. Another knowledge gap was seen in the lack of knowledge of the concept; “my five moments for hand hygiene” [13]. While participants did affirm that they practiced HH during certain of the flag posts, it was more out of habit and self-protection and not necessarily due to sound knowledge.

This is in keeping with the work by Whitby where they found that HH habits are developed before adolescence and that by adult life most persons are already set in their ways [14]. Also, while many spoke of the use of ABHR, there was little knowledge of exactly how much of this is required for HH. If the amount is inadequate, it means hand hygiene with ABHR is just as good as using ordinary soap and water [15] and HCW may be erroneously under the impression that their HH practice is adequate. Furthermore, considering the perspective of motivation, the awareness of the importance of HH is now higher than ever before with the regular outbreaks of disease. The last ebola virus disease outbreak in West-Africa spread to Nigeria in July 2014 [16]. A study by the centre for public policy alternatives (2014), during the outbreak showed that HH practice within the Lagos metropolis was very high [17]. This improvement was seen in both HCW and non-HCW. This awareness of the disease outbreaks, which could serve as a bedrock for motivational efforts at HH, has not been utilized in Adeoyo Maternity Hospital. Awareness is not enough as it must be followed up with active measures to improve performance because poor HH practice may be widespread even in the face of a high knowledge of risk factors and precautionary measures against the disease [18]. While some attested to having attended one seminar or the other, for the majority, there was no sense of an organizational effort to articulate the need for improved HH practice. The poor remuneration of staff and general disaffection with the authorities were pointers to the low morale of workers who desired to put in their best but had enormous challenges stacked against them.

A common theme that was present throughout the study was the frequent lack of tools needed to carry out HH. Information and even motivation are not enough to ensure adequate HH practice if there are lack of tools, skills and strategies. Inadequate wash stations, frequent shortages in potable water supply and power outages often impeded practice. Such challenges are all too common and have been reported before. Segun reported lack of running water and non-availability of ABHR as reasons for poor HH in a teaching hospital in Nigeria [11]. A rural hospital in India also found similar lack of resources as a cause of poor HH [19]. In the case of ABHR, its supply may need to be regular because if it is to be used adequately, one single episode of HH performance may require up to 2ml of ABHR [20]. In a setting where portable sanitizers often come in 50ml containers, adequate HH performance will require a huge investment in resources. The glaring lack of awareness of official regulations is another area that shows a deficit in behavioural skills. Besides the occasional poster on the wall promoting practice, there were no methodical and well thought out regulations. Sustained improvement in HH among HCW and reduction in methicillin resistant *S. aureus* transmission rates observed by Pittet [21] shows that vague initiatives that are not even understood by the people they are meant to mobilize is not enough. Rather improvement in HH can only be achieved through a well-articulated program. Our study had limitations. It is a single site urban study in a secondary hospital and its findings may not be generalizable to other hospitals in Nigeria. An industrial action by HCW in this hospital affected the number of recruited participants; only 19 of 20 interviews could be carried out.

## Conclusion

In conclusion, while many HCW know about HH and self-reported compliance towards it seems to be high, knowledge gaps, poor practices and inadequate ABHR use among HCW were observed. Lack of resources, absence of regulations and poor working conditions were impediments to the successful implementation of HH practices. We recommend for hospitals to institute well-articulated HH regulations and continuous education and training of HCW. Hospitals should also ensure adequate provision of resources for HH and institute a continuous monitoring and feedback program to evaluate compliance with regulations. Again, legislation mandating hospitals to set up infection control units with adequate budgetary allocation for their operations should be passed.

### What is known about this topic

- Poor compliance of healthcare workers to hand hygiene practices in low- and middle-income countries;
- Lack of systems, poor infrastructure and limited behavioral change interventions are among that influence the poor compliance to hand hygiene.

### What this study adds

- This study sought to understand the perceptions of Nigerian health care workers towards hand hygiene and the use of ABHR. To the best of our knowledge, the study fills a gap in current research on the subject being the first qualitative study of its kind in the country;
- Disease outbreaks were seen to influence the practice of hand hygiene in general and the use of ABHR;
- Although participants rated themselves high on the practice of hand hygiene, a substantial knowledge gap on hand hygiene practice was demonstrated in this study including variation in ideas about the role and use of ABHR in hand hygiene.
